# Set Up of an Automatic Water Quality Sampling System in Irrigation Agriculture

**DOI:** 10.3390/s140100212

**Published:** 2013-12-23

**Authors:** Emanuel Heinz, Philipp Kraft, Caroline Buchen, Hans-Georg Frede, Eugenio Aquino, Lutz Breuer

**Affiliations:** 1 Institute for Landscape Ecology and Resources Management, Research Centre for BioSystems, Land Use and Nutrition (IFZ), University Giessen, Heinrich-Buff-Ring 26, Giessen 35392, Germany; E-Mails: philipp.kraft@umwelt.uni-giessen.de (P.K.); hans-georg.frede@umwelt.uni-giessen.de (H.-G.F.); lutz.breuer@umwelt.uni-giessen.de (L.B.); 2 International Rice Research Institute, Los Baños 4030, The Philippines; E-Mail: e.aquino@irri.org

**Keywords:** automatic water quality sampling system, rice paddy, nitrate, stable water isotopes

## Abstract

We have developed a high-resolution automatic sampling system for continuous *in situ* measurements of stable water isotopic composition and nitrogen solutes along with hydrological information. The system facilitates concurrent monitoring of a large number of water and nutrient fluxes (ground, surface, irrigation and rain water) in irrigated agriculture. For this purpose we couple an automatic sampling system with a Wavelength-Scanned Cavity Ring Down Spectrometry System (WS-CRDS) for stable water isotope analysis (δ^2^H and δ^18^O), a reagentless hyperspectral UV photometer (ProPS) for monitoring nitrate content and various water level sensors for hydrometric information. The automatic sampling system consists of different sampling stations equipped with pumps, a switch cabinet for valve and pump control and a computer operating the system. The complete system is operated via internet-based control software, allowing supervision from nearly anywhere. The system is currently set up at the International Rice Research Institute (Los Baños, The Philippines) in a diversified rice growing system to continuously monitor water and nutrient fluxes. Here we present the system's technical set-up and provide initial proof-of-concept with results for the isotopic composition of different water sources and nitrate values from the 2012 dry season.

## 1. Introduction

Rice (*Oryza sativa L.*) is the primary food source for nearly half of the World's population, including most of the poor, and is the only cereal that can grow under wetland conditions. Most rice (0.79 million km^2^), is grown in irrigated lowlands, followed by rainfed lowlands (0.54 million km^2^) and smaller portions grown under flood prone (0.11 Mio km^2^; deep water rice) and rain-fed upland conditions (0.14 Mio km^2^; non-flood rice) [1]. Water availability is a major concern in present and future food production, by 2025, 0.22 million km^2^ of dry season rice may suffer from “economic water scarcity” [2].

Attention has to be drawn towards the best ways to manage water and nutrients to increase food production without raising the consumption of freshwater resources. New ways of fertilization need to be developed to find possibilities for resource savings and to increase yields. Site-specific nutrient management approach is such a tool to achieve improved plant nutrition and higher yields [3,4].

Water-use in rice fields has been investigated by various researchers. Tuong and Bhuiyan give an overview of the possibilities for improving water-use efficiency for rice production at the farm scale [5]. Aerobic rice systems are a key approach to increasing water-use efficiency. However, current mean yields are 32% lower in the dry season and 22% lower in the wet season in comparison to flooded conditions, wetter soil conditions in the wet season aerobic plots are the main reason for higher yields in comparison to dry season aerobic plots [6]. Rice-maize crop rotations are another management option to counteract increasing water scarcity and to assure food security, whereby the impact of intense rice-maize systems on long-term profitability and environmental factors like greenhouse gas emissions, soil quality and fertility are largely unknown [7]. A more detailed understanding of water and nutrient cycling in rice-based cropping systems is needed, and requires the investigation of hydrological and biochemical processes as well as transport dynamics at the field scale.

High-resolution spatial and temporal data are crucial to improving our understanding of environmental turnover processes in ecosystems such as mineralization, nitrification and denitrification. They reveal information which is normally lost due to infrequent or geographical strewn sampling. Kirchner *et al.* used the easily acquired parameters discharge, conductivity and pH to provide insight into the benefit of high-resolution measurements [8]. Especially at the interface of terrestrial and aquatic ecosystems biogeochemical fluxes change rapidly. Flooded rice fields are such systems where varying aerobic and anaerobic conditions impact carbon and nitrogen turnover. The lack of process understanding of these phenomena is a barrier to accurately modeling biogeochemical cycles across spatiotemporal scales [9]. In irrigated agriculture, high-resolution data on irrigation amounts and groundwater table depths and/or concentrations of solutes such as salt, total nitrogen, nitrate and ammonium provide valuable information to analyze water and fertilizer dynamics in cropping systems. Apart from simple hydrometric measurements of water level or discharge, stable water isotopes have gained importance in hydrological research in the past years. They allow the tracing of relevant water exchange and transport processes in the soil-plant-atmosphere domain [10–12].

Automatic measurement systems increase the potential for high temporal resolution sampling at a given spatial dimension and allow inter-comparison of systems and processes without increasing the expenses for analytical equipment. For example, Butterbach-Bahl *et al.* developed an *in situ* automatic measurement system for the analysis of trace gas fluxes at multiple sites [13] and Breuer *et al.* further refined the system into a mobile set-up [14]. Automatic sampling systems for water, due to the cost of analytic devices and the high energy demand for water transport, are usually situated in order to perform sampling at single locations such as streams or groundwater. Alternating sampling of different sources as in the aforementioned gas sampling system is scarcely needed because water quality in most cases does not change within a few meters. However, rice cropping systems show high variability in water and nutrient management within small distances [15], such that dense spatial water monitoring could be helpful in investigating multiple cropping systems with a single analytical system.

New developments in analytical devices permit monitoring parameters at temporal resolutions recently impossible/cost prohibitive. These new systems facilitate high-resolution data acquisition without much necessary maintenance or analysis over longer periods. For example, recent developments in laser-based spectroscopy (e.g., Wavelength Scanned Cavity Ring-Down Spectrometry-WS-CRDS; Off-Axis Integrated Cavity Output Spectroscopy-OA-ICOS) allow measurements of gas isotopic signatures *in situ* at relatively low cost without use of chemicals.

Bai *et al.* used such a laser spectroscope to measure the flux of ^13^CO_2_ in up to 48 sample vessels to determine biodegradation and extra carbon amendment to soils [16]. Various accessories for continuous site/specific water analysis were also developed and tested. Berman *et al.* modified an OA-ICOS liquid water isotope analyzer for rapid sampling and included a stream and precipitation sampling system in an auto-sampler for continuous measurements [17]. Koehler and Wassenaar utilized an equilibrator to bring water samples into the gas phase and then analyze them with a WS-CRDS [18]. A microporous hydrophobic membrane contactor was coupled to a WS-CRDS for high-resolution water measurements by Herbstritt *et al.* [19]. Munksgaard *et al.* developed a sampling device for continuous water analysis with WS-CRDS using diffusion through porous polytetrafluorethylene (PTFE) tubing [20].

Stream solutes are typically grab-sampled or taken by automatic sampling devices and then analyzed in the laboratory. Alternatively, portable probes for *in situ* detection such as ion-selective probes can be used directly in the field. These probes are practical for studies where higher detection limits are necessary and frequent site visits are feasible [21]. Recently, developments in new hyperspectral UV photometers have resulted in small and reagent-free systems for water analysis at low detection limits. With these new instruments it is now possible to observe the closely related hydrological and biogeochemical fluxes of water, C and N as proposed by Chen and Coops [22]. Sandford *et al.* showed the general utility of a hyperspectral UV photometer in surface water application for analyses of nitrate and nitrite [23]. Others have used similar instruments to measure dissolved organic carbon (DOC) concentrations in bog streams [24] and blackwaters of the Amazon [25]. In principal, such instruments can detect these C and N solutes at the same time [26].

Here we present a new type of automatic sampling set-up that facilitates *in situ* analysis of hydrometric information, stable water isotopes and nitrate concentrations in spatially differentiated agricultural fields. As proof-of-concept the system is installed in differently managed rice paddies. After an in-depth description of the sampling setup we present the software we have developed for remote control of the system via the Internet. We conclude with a number of example measurements.

## 2. Materials and Methods

### 2.1. Experimental Sites

This work is part of the interdisciplinary and transdisciplinary Research Unit “Introducing Non-Flooded Crops in Rice-Dominated Landscapes: Impact on Carbon, Nitrogen, and Water Cycles” (ICON) which focuses on the ecological impact of future changes in rice production in Southeast Asia. The principal objective is to gain necessary process understanding that will facilitate maintenance of fundamental ecosystem services, decrease environmental impacts and increase the output of high-yield cropping systems. For this purpose the effects of altered flooding regimes (flooded *vs.* non-flooded), crop diversification (wet rice *vs.* dry rice *vs.* maize) and fertilization strategies (N fertilization) are observed. The focus is to improve knowledge of the biogeochemical cycles of carbon and nitrogen, greenhouse gas emissions, the water balance, and other important ecosystem services of rice-cropping systems. For this aim a field experiment was established at the lowland farm of the International Rice Research Institute (IRRI) in the Philippines.

**Figure 1.**
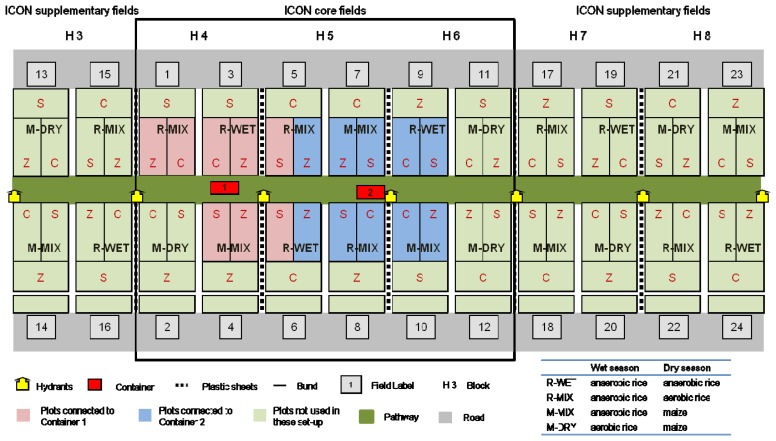
ICON experimental field design with core and supplementary fields. Each contain twelve fields with four crop rotations triplicated and three different nitrate fertilizer treatments (Z = zero-N, S = site-specific and C = conventional). Two containers house the sampling system and analytical devices. Pink shaded fields are connected to the system in container one and blue fields to the system in container two. Fields are separated by bunds or plastic sheets inserted 0.3 m into the ground.

The experiment was planned as an extended Before-After Control-Impact Design [27] with time schedule and randomized block design. During the “Before” phase all fields were equally managed as flooded rice. As “Impacts” in the “After” phase the crop rotations R-MIX (flooded rice–dry rice), M-MIX (flooded rice–maize) and M-DRY (dry rice–maize) were established. As a “Control” in the BACI design R-WET (rice flooded–rice flooded) was continued. Each field includes plots with conventional, site-specific and zero nitrogen fertilization. The experiment design contains a systematic triplication of the experiment fields. A total of twelve fields (IRRI blocks H4-H6) with 36 plots were included in the core sample design (Figure 1). The field sizes are between 530 and 550 m^2^ and the plot sizes vary between 160 and 167 m^2^. Supplementary fields (blocks H3, H7, H8) were included for destructive field sampling. Irrigation water was supplied by a hydrant system located in block H1 with block H2 as a buffer zone between the water reservoir and fields. The total area used for the core and supplementary fields (without buffer zone) is about 150 × 100 m. Analytical instruments were housed in two containers on the path between the core fields.

Sampling equipment was set up in the 18 plots closest to the path and containers. The outer plots were excluded due to requirements for flushing the sampling system prior to analysis. Each management and fertilization combination was included twice, with the exception of M-DRY. Because of the unlikelihood of remunerative cultivation of maize in wet seasons on lowland sites the M-DRY rotation was omitted.

The rice variety NSIC Rc222 was grown on anaerobic (flooded) fields, with variety NSIC Rc192 and maize variety Pioneer P3482YR on aerobic fields. All fields were irrigated (maize and non-flooded rice) or flooded (wet rice) up to two weeks before harvest. A total of 130 kg·N·ha^−1^ urea was applied over three fertilization dates (30/50/50 kg·N·ha^−1^) per conventionally fertilized plot. Each site-specific fertilized field received three fertilizations (45/60/75 kg·N·ha^−1^) on different dates, totaling 180 kg·N·ha^−1^. Plant nitrogen requirements for site-specific fertilized fields were determined with the IRRI leaf color chart [28].

### 2.2. Sampling Setup

**Figure 2.**
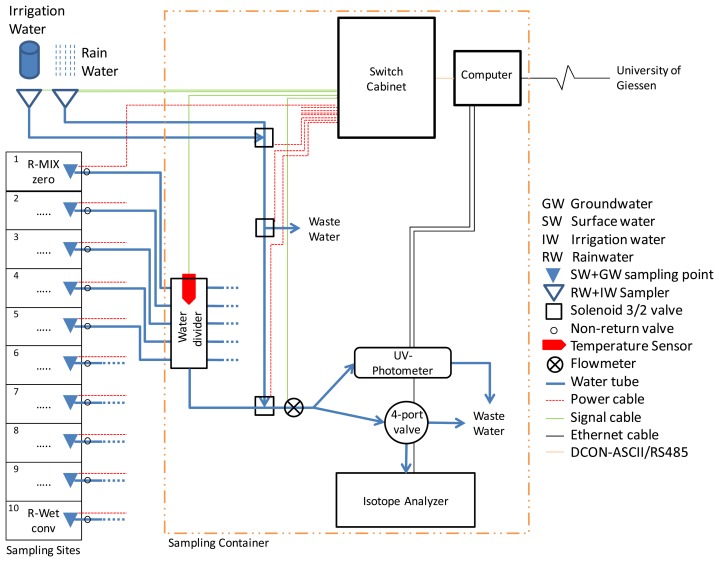
Technical set-up of the automatic water sampling system (container two) with a subset of ten plots: three water management schemes (R-WET, R-MIX and M-MIX) and three different fertilization practices (zero-N, site-specific and conventional), equipped with sampling stations for GW and SW sampling. These sampling stations provide a sample stream to the analytical equipment for *in situ* measurements of stable water isotopes (L2120-*i* Picarro) and nitrate (ProPS UV).

Challenges in this experimental setup include enabling automatic sampling of water of different sources (surface, ground, rain and irrigation water) and transporting these samples a rather long distance to the analytical equipment for direct analysis in two containers (Figure 2). We therefore constructed a sampling system consisting of 18 sampling points for groundwater (GW) and surface water (SW), additional sampling points for irrigation water (IW) and rain water (RW), a water transport and distribution system including a remote-control computer, a switch cabinet and several analytical devices.

### 2.3. Sampling Points

#### 2.3.1. Groundwater and Surface Water

**Figure 3.**
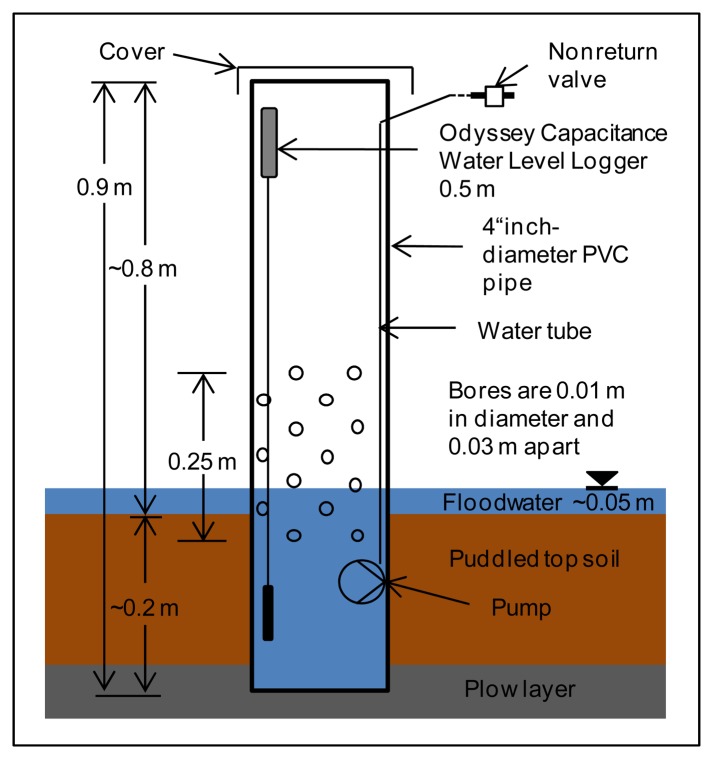
Surface water sampler for water sampling and monitoring of water depths in rice paddies. Each surface water sampler is equipped with a sampling pump, nonreturn valve and capacitance water logger.

**Figure 4.**
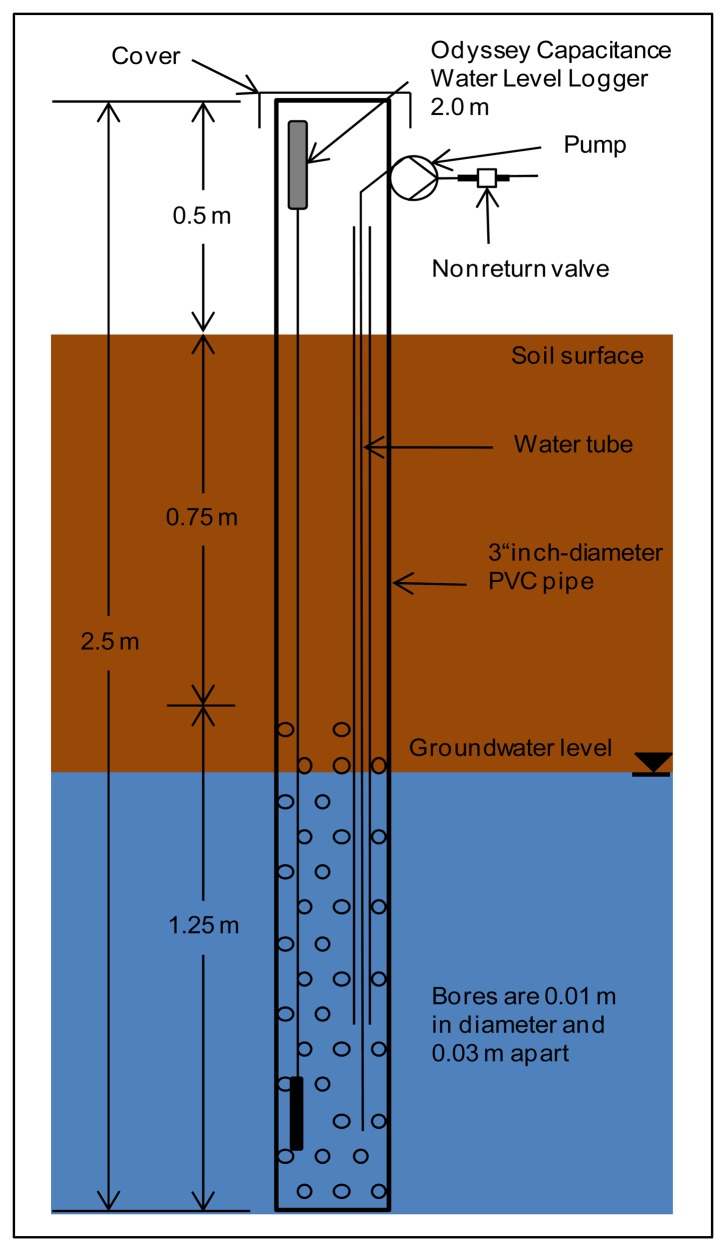
Groundwater piezometer for water sampling and monitoring of groundwater table depths. Each piezometer is equipped with a sampling pump, nonreturn valve and capacitance water logger.

Two types of water sampling stations were constructed, one for SW sampling and the other for GW. The SW sampling points (Figure 3) are made of common 4″ PVC pipe with a length of 0.9 m, perforated with 0.01 m holes, spaced 0.03 m apart, over a length of 0.25 m starting 0.15 m from the bottom of the pipe. The GW sampling points (Figure 4) consist of common 3″ PVC pipe of length 2.5 m, with the same perforation as above, over 1.25 m, starting at the bottom. The top and bottom of the pipes were closed with caps to prevent transpiration and contamination. The perforations were wrapped with jute cloth to prevent clogging by soil sediment. The SW sampling stations were installed 0.2 m deep in the soil to measure standing water in the fields. The GW sampling stations were installed upright with the bottom 2 m below ground. The space between GW sampling stations and soil was filled with gravel (approximately 0.5 m below ground) and sealed with clay.

Sampling points were equipped with capacitance loggers to record water levels (Water Level Capacitance Loggers, Odyssey Dataflow Sys, Christchurch, New Zealand) using 2 m sensor lengths for GW and 0.5 m sensor lengths for SW measurements. The logger has a resolution of approx. 0.8 mm and a memory capacity of 64 kilobytes. Water levels are recorded every 15 min. Data is downloaded manually and sensors are cleaned once a month. Every third GW and SW sampling station is additionally equipped with a water temperature sensor (KP-PT100-4.0-3L-w, Temperature Control GmbH, Donaueschingen, Germany) and connected to the analogue input in the switch cabinet.

#### 2.3.2. Rainwater and Irrigation Water

For RW sampling a funnel with 0.3 m diameter was mounted on top of a container and a second funnel for IW sampling was installed alongside. The funnel for IW was covered and connected to a pump. RW flows into the sample line by gravity. Both funnels are connected to the sample line via three 3/2 solenoid valves (Figure 2). The first solenoid valve (RW/IW valve) is connected to the two funnels to switch the sample source between RW and IW and forward these to the next valve. With this second solenoid valve (waste valve) the water from the two funnels can be discarded if it is too old or no longer required. Otherwise the sources are connected to the third solenoid valve. The third solenoid valve (sample line valve) connects the field sample line or the RW/IW water sample line to the final sample line that feeds the analytical equipment.

The solenoid valves are wired to the digital output card in the switch cabinet. To trigger RW measurements a tipping gauge is installed next to the RW funnel at the roof of its container and the IW pump is triggered manually with a push button. Both these pulse emitters are connected to the digital input cards included in the switch cabinet.

### 2.4. Transport and Distribution of Water Samples

The main challenge in the given set-up is finding a way to automatically transfer water samples from multiple sources to the analyzers. It was decided to use low-cost 12 V DC windshield-wiper fluid pumps (OE number 8377612, various manufacturers) from auto supply stores at each sampling point in the field. These pumps are powerful enough to pump 40 L·h^−1^ over a distance of more than 30 m through Polyethylene tubes (inner diameter 6 mm) and still provide a strong water stream to flush the tubing and system in a short time to enable a high sampling interval. Moreover, they are cheap and available worldwide. The downside of these pumps is that they do not draw water hence must always be in contact with water—a problem at some sampling points in rice paddy systems with their dynamic water levels. The water volume sampled is measured and recorded with a digital flowmeter (FCH-M-POM-LC (G 1/8), B.I.O.-Tech, Vilshofen, Germany) at the end of the sample line in the container (Figure 2).

The pumps in the piezometers are installed at the top off the pipes for easier maintenance. To prevent false readings by the capacitance water level loggers, the tubing in contact with the GW is surrounded by a slim pipe. The SW pumps are covered with a filter sponge to prevent suction of sediment and placed directly inside of the SW sampler at the bottom of the pipes. The pumps are connected with tubes to a divider inside the container, equipped with nonreturn valves to prevent loss of a continuous water line between GW and pump and pumping water from one source to another. To provide power and for control, the pumps are connected to the switch box. Two different types of power cables are used to connect the pumps to the switch box depending on the distance to the container. To provide a current of at least 2 A cables with 1.5 mm^2^ for distances under 20 m and 2.5 mm^2^ for longer distances were used. Cables were connected with cable shoes to the pumps and sealed with hot glue for water resistance. The distance from sampling station to container varied between 7 m and 26 m.

### 2.5. Switch Cabinet

**Figure 5.**
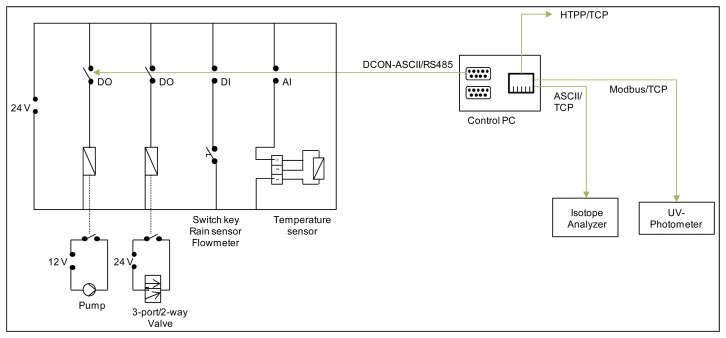
Schematic circuit diagram of the switch cabinet including analogue input, digital in- and output cards with connections to pumps, valves, push button, rain sensor, flowmeter and temperature sensors as well as a connection to the control PC and connections from the control PC to the analytical equipment and the internet.

The switch cabinet (Figure 5) is the link between the field equipment, pumps and temperature sensors, and the control computer. The switch cabinet contains a surge protector, connected to a permanent power supply, 12 V and 24 V power transformers. The 24 V transformer provides electricity to an input/output (I/O) casing containing analogue input, digital in- and output cards (RU-87P8-G-CR, I-87015PW-G CR, I-87051W-G-CR, I-87057W-G-CR, Spectra Computersysteme GmbH, Reutlingen, Germany).

Digital output cards are responsible for the pump and solenoid valve control. The analogue input card is connected to temperature sensors. The digital input card receives signals from the flowmeter and the tipping rain gauge. The I/O casing is connected to the control computer via a RS-232 to RS-485 converter and serial PC-cable. The 12 V transformer provides power to the pumps and solenoid valves, with relays used to start a pump or change the flow path of a valve.

### 2.6. Sample Analysis

#### 2.6.1. UV Process Photometer

For the analysis of nitrate in water we use a hyperspectral UV-process photometer (ProPS-WW, Trios, Rastede, Germany). The system is small (68 mm diameter × 520 mm) and portable (5 kg). It uses a 5 W Deuterium lamp which has a stability of under 0.1% in a few seconds. The system records spectra between 190 nm and 360 nm with a pixel distance of 0.9 nm. This allows to differentiate between nitrate and nitrite. These spectra are simultaneously analyzed for nitrate with mathematical analysis software installed on the Tribox2 control system (Trios, Rastede, Germany). Additional sampling parameters like DOC or nitrite can be added if desired. The ProPS has an adjustable optical path length between 1 and 10 mm, with a detectable nitrate range at 10 mm between 0.07 and 3.65 mg·L^−1^ NO_3_-N for field conditions. Sampling intervals can be freely selected, but should not be set under 900 s for longer periods as the deuterium lamp can be damaged or its lifespan rapidly reduced. The general applicability of these sensors for SW monitoring has been shown by Sandford *et al.* [23]. A 1,800 s sampling interval was used in accordance with the sampling interval of the Cavity Ring Down Spectrometry System (see Section 2.7). The ProPS can be submerged or used with a flow-through cell and runs reagent-free. The ProPS probe was installed with a flow-through cell directly connected to the sample line to allow sampling for a number of different sources. Both containers (Figure 2) are equipped with a ProPS probe. The optic is cleaned twice a week with acetone and a tissue by a local fieldworker to ensure a clean optical path. Standard addition method was carried out with GW and comparison measurements were made in the Analytical Service Laboratory of IRRI (y = 0.98× + 0.14, R^2^ = 0.99). Nitrate concentrations were of the same magnitude as the ProPS values.

#### 2.6.2. Cavity Ring Down Spectrometry

New developments in portable laser spectroscopy systems have revolutionized the direct quantification of trace compounds and isotopes in biogeochemical research. Here we use a Wavelength-Scanned Cavity Ring Down Spectrometry system (WS-CRDS) (L2120-*i*, Picarro, Santa Clara, CA, USA) for δ^2^H and δ^18^O analyzes with a high precision vaporizer (A0211, Picarro, Santa Clara, CA, USA). WS-CRDS has been shown to measure stable water isotopes in the same accuracy as high-temperature conversion isotope ratio mass spectrometry (TC/EA-IRMS) [29]. Dry gas (air or N_2_) is required as a transport gas for the WS-CRDS system, other reagents are not necessary. The L2120-*i* for water isotopes can analyze air quasi-continuously, while liquid water is usually analyzed from vials connected to an auto-sampler. Samples are vaporized with either a high throughput vaporizer in 300 s or with a high precision vaporizer in approximately 540 s intervals. The latter approach was used in this study. It is recommended to measure at least three replicates to calculate the isotopic values of a sample. The first two measurements are discarded and the following are used for quantitative analysis to avoid carry-over effects.

Instead of running an auto-sampler and analyzing batch samples, it was decided to use a 4-port valve (Internal Sample Injector, VICI Valco, Schenkon, Switzerland) driven by a pneumatic actuator with a 2 μL sample loop for automatic and direct sampling of water aliquots from the sample line. The 4-port valve is part of the sample line and provides water to the vaporizer every 540 s for quasi continuous water analysis. The valve runs like the analyzer with N_2_ for the position change of the valve and for the injection of the sample to the vaporizer. The 4-port valve is embedded in a custom made system (Meteorology Consult, Königstein, Germany) consisting of a further pump, mechanical parts, a N_2_ pressure controller and a water flow controller. Control software (PicAdam; Meteorology Consult, Königstein, Germany) is installed on the Picarro L2120-*i* that triggers the time intervals for flushing, the position change of the valve sample loop and the injection of the water sample into the vaporizer.

### 2.7. Controlling Software

The whole system is controlled by software developed in-house for this study, running on an industry PC (NISE 101, SEPCTRA, Reutlingen, Germany) with the Xubuntu operating system. The controlling software is written in Python as an object-oriented, asynchronous and modular framework using various open source Python packages from the Python package index [30]. As such, the software is portable to different platforms and available on request from the authors under an open source license agreement. The self-developed modules are responsible for the scheduling of sampling, communication with the valve/pump system and the analytic devices using several bus systems, protocols and a web server to serve as the user interface.

The valve/pump system is controlled in the switch cabinet using a serial connection over a RS232 port with the ASCII based DCON protocol. The DCON module of the software provides an interface for asynchronous communication using the DCON protocol using the python package “serial”. The I/O modules, digital in- and outputs and analogue temperature input are available as software elements for the other modules. The interface provides classes as relays, counters (for rain gauges and flowmeters) and temperature sensors used by the sampling sequence module. A MODBUS-TCP connection is used for triggering measurements and retrieving measured data from the UV photometer. The core functionality is wrapped by a specialized module using the “pymodbus” package.

The WS-CRDS provides direct communication over a simple ASCII Telnet protocol. However, this protocol is designed for continuous gas measurements and not for the more complex cycle of sample injection, evaporation, measurement and cavity drying used for liquid water analysis. Since the control software of this sequence is written in Python by the manufacturer and is partly accessible, the software was extended by the authors to write the sequence status not only on the screen but also over a standard Ethernet connection to an open IP socket of the control software. The control software reads the status messages and schedules the sample-taking mechanism according to the measurement status of the isotope sample injection cycle. If protocols are changed, only the low level modules need to be adapted to these changes. For off-site software tests, virtual simulations of the hardware are available.

**Figure 6.**
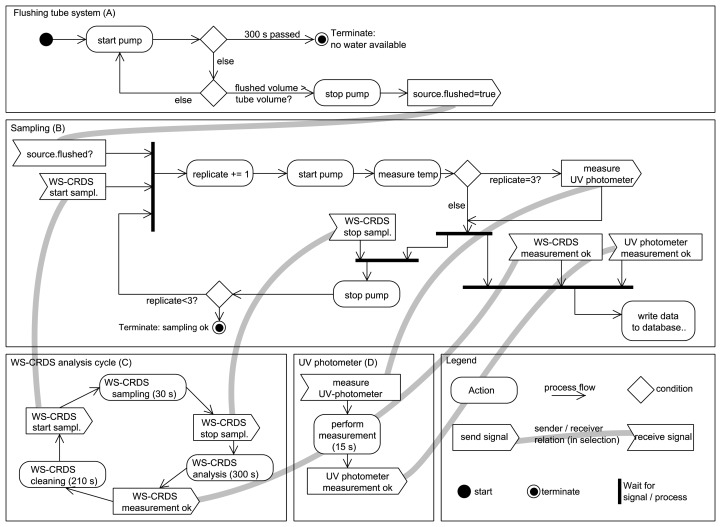
UML2 activity diagram of the sampling sequence with isotopic analysis. Each communication with external devices is asynchronous to check for errors or non-responding devices. Additional checks to preserve the pumps from running dry are omitted in the diagram. Non-responsive modules/devices are checked with time-out constraints (not shown for simplification).

The sequence of processing a single sampling is assembled using the high-level interface for the hardware devices. This module is read from the hard disk before execution to allow online changes of the sequence without the need to restart the main program. The sampling sequence with isotope analysis is depicted in (Figure 6). The first part of the sampling sequence (A) flushes the tube system connecting the water sources (GW, SW, RW, IW) with the analysis equipment to ensure that water from that intended source is measured. The pumped volume is recorded and the tube system is flagged as “flushed” if the pumped volume exceeds a user-given tube volume. The second part (B) contains the sampling for the isotopic system. As mentioned earlier, the ring down cavity of the WS-CRDS needs to be flushed with water vapor at least two times. The sampling sequence waits for the WS-CRDS injection system to pump fresh water from the source during the injection time (approx. 30 s) (C). The water temperature of the sample is measured at the analysis devices, and of the water temperature at the intended source, if it is installed with a temperature sensor. The photometer measurement is triggered only during the third replication of the WS-CRDS injection, to preserve the performance of the Deuterium lamp (D). After completion of the sampling, a new asynchronous job is started to wait for the completion of the measurements (*ca.* 15 s for UV photometer and *ca.* 300 s for the WS-CRDS) and to store the results in a SQLite database (E). During that time, the sampling sequence proceeds, e.g., by flushing the tube system with water from the next scheduled source or waiting for the next sampling of the WS-CRDS. Errors reported during any of the tasks are logged in the database for later review but do not stop the sampling sequence. In case of fatal errors, like hardware malfunction in the pumping system or missing water, the sampling of the current source is terminated. If only UV photometer measurements are performed, the sampling sequence is not repeated and a user-defined waiting period between the measurements (e.g., 1,800 s) is used.

The sampling of the water sources from the fields (GW, SW) are scheduled by a user-defined queue and adopted to the actual measurement, e.g., during the dry season. Surface water is only measured on fields with flooded rice. However, the scheduling system is “listening” for certain events to adopt the given sampling schedule. During rain events (detected by the tipping gauge), RW sampling is put to the front of the queue of waiting sample sources so that it handled directly after the current sample source. RW analysis is performed if more than 4 mm of water has collected in the RW funnel and no RW measurement has been carried out in the last 8 h, to avoid endless loops when long rain events occur. A similar mechanism is used to sample IW, triggered by a push button outside of the container to be pressed by field workers after filling IW in the sample collector. Both RW and IW collectors are emptied after sampling.

A webserver (“cherrypy” and “genshi” packages) enables users managing the whole system, via a field laptop onsite or over a GPRS connection offsite, to start/stop the sampling schedule, change the sequence of sample sources and access the actual measured data and the automated system logbook. Measured values are plotted as timeline graphs on the system webpage using the “matplotlib” package.

## 3. Results

The following sections show the overall functionality and a proof-of-concept of the system. The aim is not interpreting the measurements in detail as much as showing the value of the high-resolution automatic sampling system.

**Figure 7.**
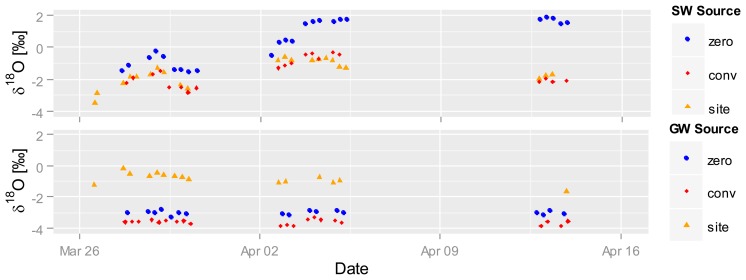
δ^18^O isotopic composition of SW (**top**) and GW (**bottom**) from different fertilized rice plots (zero, conventional and site-specific) between the end of March and mid-April 2012. Gaps in the graph are given due to setup changes and system testing. Note that only the instrument's internal calibration was used.

Figure 7 shows automatically recorded δ^18^O values in the SW (top) and GW water (bottom) between end of March and mid April 2012 (dry season, 58 to 76 days after seeding) for selected plots. Gaps in the graph are due to setup changes and system testing. The δ^18^O values in the SW plots increase from 26 March on, starting at −3‰ and reaching almost −1‰ on conventional as well as site-specific plots, and from −2‰ to 0‰ on zero-fertilized plots. A sharp drop on 28 March occurs on all sites; δ^18^O values then steadily increase until 5 April. While δ^18^O values in the zero-fertilized plots stay at the same level of 2‰ until 14 April, they fall back to the base level in the conventional and site-specific fertilized plots. We speculate that the richer δ^18^O signatures in the SW of zero-fertilization plots are related to less shading by plants due to weaker plant growth and stronger evaporation. The other two fertilized plots show stronger plant growth and higher shading and are therefore less enriched. Contrastingly, δ^18^O signatures in the GW stay relatively constant the whole time (site-specific 0‰–1‰, conventional −3.5‰, zero −3‰), indicating no direct interaction between SW and GW during this period of time. The value of the high frequency, *in situ* sampling system is depicted by the detailed record of the changes in δ^18^O in SW—a dynamic that would not have been captured by conventional, less frequent grab sampling.

**Figure 8.**
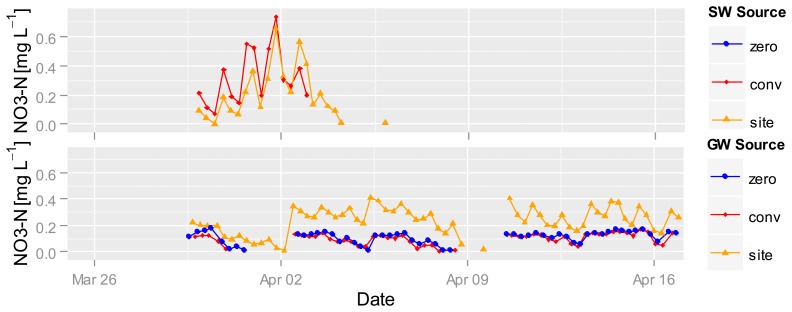
Nitrate concentration in SW (**top**) and GW (**bottom**) in different fertilized rice plots from 30 March to 17 April. Nitrate concentration in zero-fertilized SW was under the detection limit of the sensor.

Nitrate measurement in wet rice fields started 30 March. Figure 8 shows the nitrate concentrations in the GW and SW of different fertilized plots. The highest nitrate values of 0.6 mg·L^−1^ NO_3_-N where recorded in SW at the beginning of April after a strong increase both at the conventional and the site-specific fertilized plots, followed by a strong decrease in nitrate concentrations down to the detection limit of the UV photometer. The nitrate concentration in the zero-fertilized SW plot was under the detection limit of the sensor.

**Figure 9.**
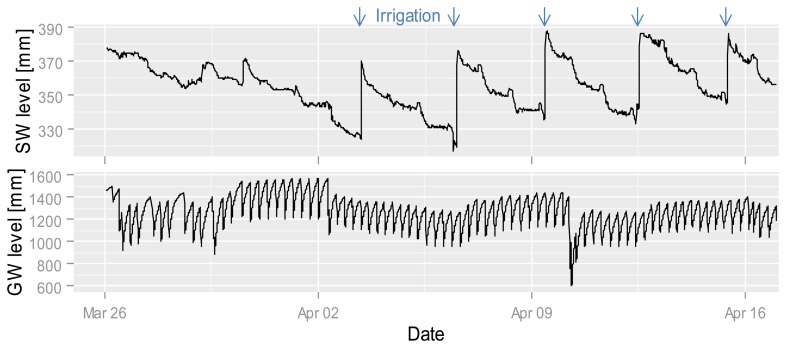
Relative SW (**top**) and GW (**bottom**) levels from the end of March to mid-April at the site-specific fertilized plot. The strong increases in SW level are irrigation events. The regular fluctuation in the GW is given due to pumping and refilling of the piezometer.

Nitrate values in the GW are almost always greater than the detection limit. The nitrate concentration in the site-specific fertilized GW is higher than the concentration under the conventional and zero-fertilized plots. The two time points (2 and 10 April) with the highest nitrate increases (despite the data gap before the 10th) in the site-specific fertilized GW plot are related to a drop in the GW level (Figure 9). The increase at this point could be caused by the onset of aerobic conditions in normally saturated soil, nitrification and transformation into the GW, whereas the first drop in GW levels is during drier conditions than the second (Figure 9). It is possible that the GW is mainly affected by soil conditions and GW flow direction so that under all plots nearly the same nitrate concentrations exist. It seems that GW is less affected by surface events, whereas high-frequency measurements enable capturing and comparing short-term differences which allow to develop hypotheses for underlying processes.

## 4. Conclusions

Frequent sampling and analysis of multiple water sources was until now a coast expansive and time intensive work. We have shown that automatic measurements of water from various sources are possible. The communication between different analyzers and the system works without problems. Further, devices for continuous water supply to the isotopic analyzers have been developed by other researchers and can be adapted to this system and an add-on module for the supply of standard solutions to the isotopic analyzer is now available by the manufacturer. Water quality sensors could also be easily integrated into the sampling sequence.

High frequency measurements provide the basis for insight into hydrological and biogeochemical interaction that could not be investigated before, especially with regard to spatially distributed sampling and where multiple water quality parameters need to be monitored. The generated data facilitates improved system process understanding and helps investigate erratic events such as the break-through of nutrients by leaching in preferential flow systems. The system presented here could be helpful in water studies where multiple sources or high-frequency measurements of unique sources are to be studied (aquaculture, irrigated agriculture, monitoring of rain events, multiple-source monitoring *etc.*).
